# Development of a highly sensitive gas chromatography–mass spectrometry method preceded by solid‐phase microextraction for the analysis of propofol in low‐volume cerebral microdialysate samples

**DOI:** 10.1002/jssc.201801066

**Published:** 2019-01-30

**Authors:** Armin Sebastian Guntner, Simon Stöcklegger, Michael Kneidinger, Udo Illievich, Wolfgang Buchberger

**Affiliations:** ^1^ Institute of Analytical Chemistry Johannes Kepler University Linz Austria; ^2^ Department for Neuroanesthesia and Intensive Care Kepler University Hospital Neuromed Campus Linz Austria

**Keywords:** brain, cerebral, microdialysis, propofol, solid‐phase microextraction

## Abstract

To date, the commonly used intravenous anesthetic propofol has been widely studied, and fundamental pharmacodynamic and pharmacokinetic characteristics of the drug are known. However, propofol has not yet been quantified in vivo in the target organ, the human brain. Here, cerebral microdialysis offers the unique opportunity to sample propofol in the living human organism. Therefore, a highly sensitive analytical method for propofol quantitation in small sample volumes of 30 μL, based on direct immersion solid‐phase microextraction was developed. Preconcentration was followed by gas chromatographic separation and mass spectrometric detection of the compound. This optimized method provided a linear range between the lower limit of detection (50 ng/L) and 200 μg/L. Matrix‐matched calibration was used to compensate recovery issues. A precision of 2.7% relative standard deviation between five consecutive measurements and an interday precision of 6.4% relative standard deviation could be achieved. Furthermore, the permeability of propofol through a cerebral microdialysate system was tested. In summary, the developed method to analyze cerebral microdialysate samples, allows the in vivo quantitation of propofol in the living human brain. Additionally the calculation of extracellular fluid levels is enabled since the recovery of the cerebral microdialysis regarding propofol was determined.

**Article Related Abbreviation**
DI: direct immersion

## 1 INTRODUCTION

Propofol (2,6‐diisopropyl‐phenol) is a commonly used intravenous anesthetic agent for inducing and maintaining general anesthesia and/or sedation in intensive care. It provides a quick effect (already after 30 s) and rapid recovery of the patient due to its lack of accumulation 1, 2. Based on its apolar character, propofol is a lipophilic drug, which is quickly metabolized predominately in the liver by oxidation of the aromatic system and subsequent glucuronidation 1, 3, 4.

Propofol as a part of total intravenous anesthesia is applied in target‐controlled infusions, which became available in the late 1990s 5. Hereby the aim is to establish intended concentrations at the target organ, the brain, rather than in blood plasma 6. The resulting levels are initially predicted either by the pharmacokinetic/pharmacodynamic model of Schnider or by the model of Marsh, which was proven to mirror more precisely the characteristic parameters sedation score and bispectral index 7. Both model's calculations are based on pharmacokinetic compartment simulations using various rate constants and are adjustable for age, body weight, and size, but none of the two models seems to date superior when applied for total intravenous anesthesia in healthy, nonsignificantly obese patients 5, 7.

Since propofol, which is also called “milk of amnesia” based on its white and milk‐like appearance as a ready‐to‐use injection solution 8, is a common anesthetic agent used in daily clinic operation, but is also a drug of abuse 8, numerous methods for its quantitation in human sample materials such as blood (serum), urine, and cerebrospinal fluid have already been published 9. They include LLE or SPE sample preparation steps followed by gas or liquid chromatographic separation in combination with UV, fluorescence, or MS (sometimes MS^2^) detection and are used intensively for pharmacokinetic and forensic research purposes 9.

Despite the fact that propofol is listed as an essential medicine by the World Health Organization and targets the central nervous system as its primary site of action 10, 11 (most probably due to its effect on the γ‐aminobutyric acid‐A receptor activity 8), to date no in vivo measurements of propofol concentrations in the central nervous system of sedated patients have been performed. However, general pharmacokinetic aspects of propofol have already been discussed in the existing literature. In particular, reference must be made to the work of Dawidowicz et al. 12, who showed the distribution ratios and levels of propofol in serum and cerebrospinal fluid samples and its protein binding in both media 11, 13. Accordingly propofol concentrations in serum are to be found in the milligram per liter range, whereas cerebrospinal fluid levels are in the low microgram per liter range 12. Protein binding of propofol is about 99% in serum and about 70% in cerebrospinal fluid 11, 13, 14.

To date, no studies have been conducted on propofol in microdialysate samples, which could provide basic information about the concentration of this drug in the target organ, due to the unique ability offered by cerebral microdialysis for in vivo analysis of human cerebral interstitial fluid 15, 16, 17. Microdialysis itself is a technique used in daily medical routine as well as medical research, where a small semipermeable membrane carried by a probe is inserted into a tissue of interest. Using a constant perfusate flow through the connected catheter tubing, it is possible to collect samples from the interstitial space and monitor neurotransmitters, metabolites of the energy metabolism such as lactate, pyruvate, glycerol, glucose, and glutamate, as well as exogenous substances such as pharmaceuticals 15, 16, 17, 18, 19, 20.

Despite its invasive character, microdialysis has become a common tool in multimodal neuromonitoring, neurointensive care, and neuroscience. In the clinical routine of neurosurgery, microdialysis is used primarily in combination with intracranial pressure measurements and the associated probe in patients with serious skull injuries and possibly increased intracranial pressure to early diagnose disturbances of metabolism and so on in the brain 21.

Besides the invasive nature and also its limited ability of resolving time related events, one issue has to be considered for each analyte—the efficacy of the diffusional process along the membrane—resulting in the recovery of the microdialysis system. In general, lowering the perfusate flow in microdialysis enhances recoveries, but validation is needed in every case 22. Most commonly used for testing recoveries of microdialysis is the so‐called zero‐net flux method, where the analyte's initial concentration in perfusate solution is steadily varied and the resulting perfusate concentration is monitored. The analyte concentration in the perfusate where no change is observable ($c_{in}=\;c_{out}$) reflects the tissue concentration and the slope of the regression line of the c in −c out  versus *c*_in_ plot reflects the recovery of the microdialysis system 23, 24, 25.

However, the zero‐net flux method is not possible in every case and another technique to access the recovery of the microdialysis system has to be used. The corresponding protocol for practical realization is described in Section 2.5 and the theoretical approach is shown below 23, 24, 25.

The amount of analyte transported by diffusion during a microdialysis experiment is described in Equation (1) according to Fick's law 18:

(1) $$J\;=\;-D\;\;\frac{\Delta c}{\Delta r}=\;\frac{\Delta c}{R_{p}+R_{m}+R_{e}}$$

*J* is hereby the flux through the membrane, *D* represents the diffusion coefficient, Δc represents the analyte concentration difference, and Δr is the distance where diffusion happens 18. The parameters *R*_p, m, e_ represent the mass transport resistances (perfusate, membrane, and external tissue), which have to be summed, just like electrical resistances in series.

To relate the measured analyte concentration in the perfusate with the actual concentration in the probed tissue, Bungay et al. 26 described a method based on transport resistance during steady‐state microdialysis introducing an extraction fraction parameter *E*_d_ 18:

(2) Ed=cp−c in ce−c in =1−exp−1QdRp+Rm+Re

*c*_p_ and c in  represent the analyte concentration in perfusate after and before (typically zero) the extraction and *c*_e_ describes the analyte concentration in the probed tissue. *Q*_d_ represents the perfusate flow rate during the microdialysis 18.

In fact, $E_{d}$ describes the efficiency of a microdialysis system, enabling the calculation of the extracellular concentration. Changes in flow rate or diffusional resistance lead to a distortion of this factor and to a decrease or increase in analyte concentration in dialysate. For practical reasons, it is generally assumed that $E_{d}$ is constant during a microdialytic sample collection, which is not necessarily valid in every case 18. Following Bungay et al., the resistanceR to mass transfer in microdialysis can be expressed as a function of geometric parameters describing the probed tissue 26:

(3) $$R\;=\;R_{p}+R_{m}+R_{e}$$

(4) $$R\;=\;\frac{\Delta r}{D_{eff}\;\varphi \;S}$$

*r* represents the distance over which diffusional processes occur. $D_{eff}$ is the effective diffusion resulting from a complex tissue geometry. φ and *S* describe volume and surface of the probed tissue, respectively, 26.

The extracellular concentration of an analyte in steady state within the body is described theoretically as 26:

(5) $$\begin{array}{ccc} \frac{\delta c_{e}}{\delta t} & = & \;D_{eff}\frac{1}{r}\frac{\delta }{\delta r}\left(r\frac{\delta c_{e}}{\delta r}\right)+k_{pl,e}c_{pl}-k_{e,pl}c_{e}+k_{r}c_{i}-\\ & & k_{u}c_{pl}-k_{m}\;c_{e}=\;0 \end{array}$$

Hereby $c_{e}$, $c_{pl}$, and $c_{i}$ represent analyte concentrations in extracellular, plasma, and intracellular compartments, respectively. The factors $k_{pl,e}$,$\;k_{e,pl}$, $k_{r}$, and $k_{u}$ describe the rate constants for the mass transport between plasma and extracellular fluid respectively extracellular and the intracellular fluid and vice versa. $k_{m}$ represents the rate constant regarding irreversible metabolism processes.r is used to express the radius of the probed volume element 26.

As the high sensitivities of SPME–GC–MS analysis methods are known and cerebral microdialysis enables in vivo sampling of human cerebral interstitial fluid, this study was intended to evaluate a direct immersion (DI) SPME method for propofol quantitation in small volume samples in terms of precision, sensitivity, and reproducibility for its use in routine quantitation in the context of extensive cerebral pharmacokinetic studies. Additionally the permeability of propofol through a microdialysis membrane was investigated.

## 2 MATERIALS AND METHODS

### 2.1 Reagents and consumables

Propofol (≥ 98 %) was purchased from TCI Deutschland (Eschborn, Germany) and was stored under nitrogen at 4°C prior to use, as recommended by the manufacturer.

Stock solutions of propofol were prepared in methanol with a concentration of 2 g/L and stored at 4°C. Calibration curves from the lower LOD (50 ng/L) to 200 μg/L were prepared in a perfusate solution (NaCl 147 mM, KCl 2.7 mM, CaCl_2_ 1.2 mM, and MgCl_2_ 0.85 mM in water, Millipore quality).

Polydimethylsiloxane SPME fibers (57300‐U, PDMS 100 μm, 24 ga) were purchased from Supelco/Sigma–Aldrich (Bellefonte, PA).

**Figure 1:**
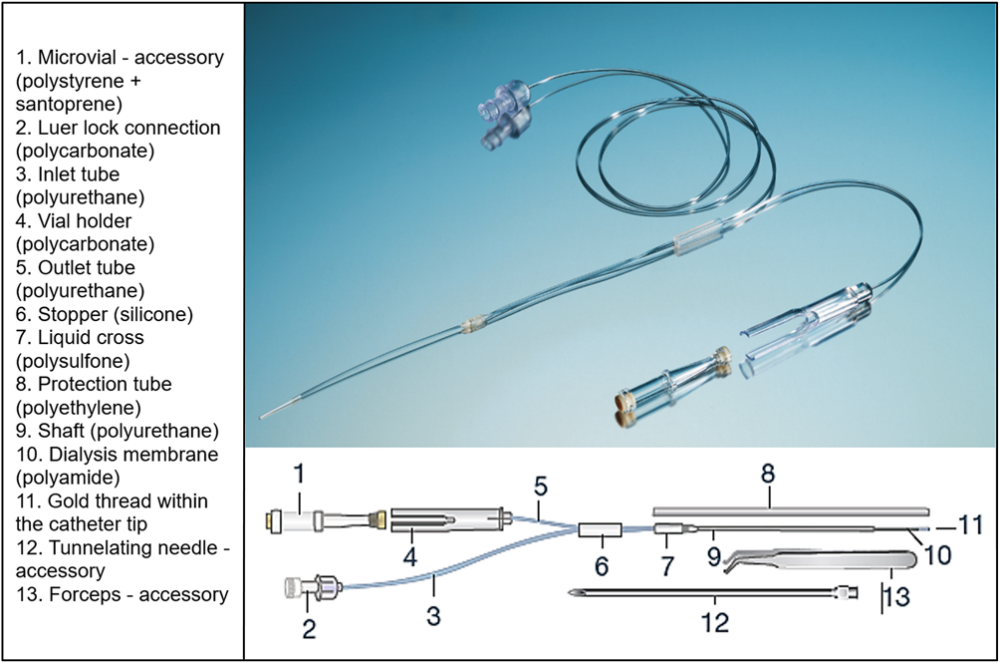
Photograph and drawing of a cerebral microdialysis catheter showing its main parts, adapted from 27

The tested 71 High Cut‐Off Brain Microdialysis Catheter (compare Figure 1) was originally distributed by M Dialysis AB (Stockholm, Sweden) 27. The catheter centerpiece, the polyamide membrane, had a molecular weight cut‐off of 20 kDa and a length of 1 cm.

Special vials (32 × 11.6 mm) with integrated 200 μL glass inserts used for the DI‐SPME were purchased from Bruckner Analysetechnik (Linz, Austria).

All further chemicals were HPLC grade and were purchased from VWR International (Darmstadt, Germany).

### 2.2 Instrumentation

GC–MS analysis was performed using an Agilent 6890N Network GC System (Agilent Technologies, Santa Clara, California) coupled to an Agilent 5973 Mass Selective Detector (Agilent Technologies, Santa Clara, California). Chromatographic separation was performed by an HP 5 MS GC Column, 30 m x 0.25 mm x 0.25 μm (Agilent Technologies, Santa Clara, California). Hereby an initial step of 60°C for 1 min was succeeded by a temperature ramp of 30°C/min up to a temperature of 225°C and a subsequent temperature ramp of 75°C/min up to a temperature of 300°C, which was held for 5 min. The mass spectrometer was operated in selected ion monitoring mode with *m*/*z* ratios for propofol detection of 117.1, 163.2, and 178.1 and included a solvent delay of 3 min and a dwell time of 10 ms. Laminar flow in in vitro microdialysis experiments for permeability testing was obtained using a Harvard Apparatus standard infusion syringe pump (Harvard Apparatus, South Natick, Massachusetts).

### 2.3 DI‐SPME preconcentration

**Figure 2:**
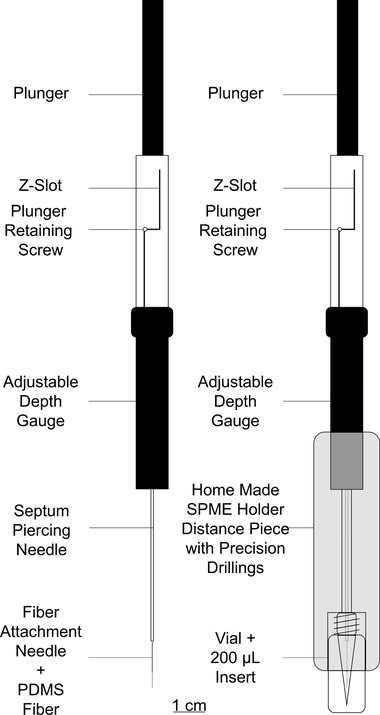
DI‐SPME with an in‐house developed distance piece for reproducible immersion depths

Since DI‐SPME is typically done with samples of larger volume, a homemade distance piece for the SPME holder was designed and constructed in‐house, to assure reproducible immersion depths of 6 mm and reproducibility of the whole analysis method in samples of 30 μL. Figure 2 shows details of the DI‐SPME system with the homemade distance piece.

### 2.4 Quantitation

Sixteen calibrators were used to check the linearity of the method in a range of 50 ng/L up to 200 μg/L. LOD and LOQ were defined at concentrations giving a signal with a S/N ratio of 3 and 10, respectively. Intraday precision was tested on the basis of five consecutive measurements of 5 μg/L propofol standard solutions and interday precision was determined as the RSD on average of six calibrators quantitated on different days. No focus was put on SPME recovery experiments since matrix‐matched calibration compensated recovery deviations. Clinical microdialysate samples lack proteins based on the membrane cut‐off of the catheter system and contain considerable amounts of salts. Therefore, the properties of the dialysate are mainly governed by these high salt concentrations and hardly by any other components originating from the interstitial fluid. This makes an external calibration in artificial perfusate a valid calibration option.

### 2.5 Recovery of cerebral microdialysis regarding propofol

For testing the permeability of the cerebral microdialysis system regarding propofol, the catheter (see Section 2.1) was immersed in a perfusate solution (NaCl 147 mM, KCl 2.7 mM, CaCl_2_ 1.2 mM, and MgCl_2_ 0.85 mM) containing 1 μg/L propofol prepared by stepwise dilution from methanolic stock solution.

**Figure 3:**
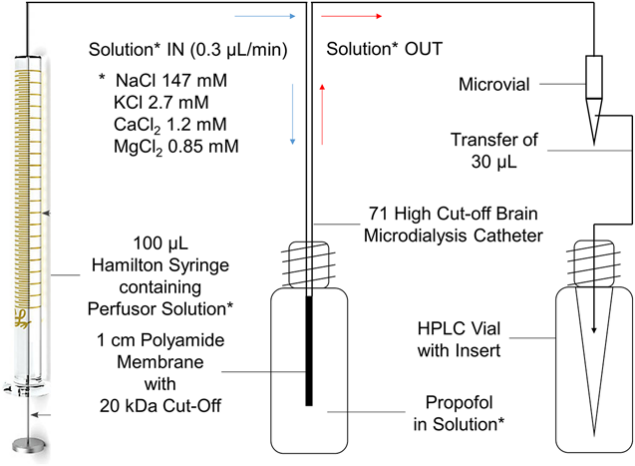
Schematic representation of the assembly to investigate the permeability of the 71 High Cut‐Off Brain Microdialysis Catheter (M Dialysis AB, Stockholm, Sweden) regarding propofol

Figure 3 shows further details of the experimental setting. A 100 μL Hamilton syringe with a Luer‐Lock connector was filled with perfusate, having a similar composition as the interstitial fluid within the human brain in terms of salt concentrations, and put on a Harvard Apparatus 22 syringe pump to obtain, after flushing (15 μL/min, 6 min), a laminar flow of 0.3 μL/min, as recommended by Ungerstedt 15. As with the microdialysis conducted in vivo on intensive care patients, every 2 h a sample of 36 μL was collected using the designated micro‐vials. Subsequently an exact sample volume of 30 μL was transferred to special vials (32 x 11.6 mm) with integrated 200 μL glass inserts and analyzed using the developed DI‐SPME–GC–MS method. The determination of the recovery of the microdialysis was performed in triplicate at room temperature.

## 3 RESULTS AND DISCUSSION

### 3.1 DI‐SPME–GC–MS

Based on the apolar and considerable volatile character of propofol, polydimethylsiloxane was the fiber material of choice for DI‐SPME 28, 29. Method development included the design of a reproducible preconcentration step followed by thermal desorption in the GC injector system. Especially the extraction time is hereby a key parameter in terms of sensitivity and time efficiency.

**Figure 4:**
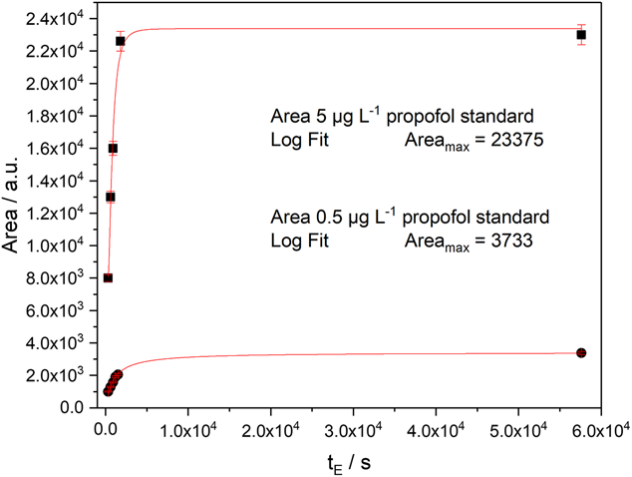
Optimization of extraction time using mathematical approximations

Extraction times of standard solutions were varied and the corresponding MS signal was monitored, leading finally to an extraction time of 30 min at room temperature. In this context, Figure 4 shows the logarithmic fitting of the data points for optimizing the extraction time. The logarithmic fit provides a good approximation of the signal versus extraction time profile of the used fiber.

Besides extraction time various other parameters of the DI‐SPME–GC–MS system were varied in order to increase signal intensities. A crucial parameter for thermal desorption and proper transfer of the analyte onto the GC column is the inlet temperature as well as the desorption time 30. One minute desorption at a temperature of 260°C showed the best results, meaning quantitative transfer of the analyte (boiling point 256°C 31) to the GC column.

The gas chromatographic separation with a run time of 12.50 min was finally conducted as follows using a helium flow of 1.5 mL/min and was based on the work of Hikiji et al. 32: An initial step of 60°C for 1 min was succeeded by a temperature ramp of 30°C/min up to a temperature of 225°C and a subsequent temperature ramp of 75°C/min up to a temperature of 300°C. The final temperature of 300°C was held for 5 min. The retention time of propofol was found to be highly stable (RSD < 0.03%) at 5.33 min under these conditions.

For further improvement of the method's sensitivity, also related parameters of the MS system were varied. The mass spectrometer was operated in selected ion monitoring mode with m/z ratios for propofol detection of 117.1, 163.2, and 178.1 and included a solvent delay of 3 min and a dwell time of 10 ms. Ionization of the analyte was obtained using electron impact ionization with a kinetic energy of 70 eV. Data analysis and calibration were done by linear regression analysis of peak areas obtained by integration of *m*/*z* 163.2 signals using MSD ChemStation Software (Version E.02.01.1177) according to the best S/N ratio of this ion in comparison to the other fragment ions. Additionally the MS detection window was set to be between 4.0 and 6.1 min for longer MS detector life.

**Figure 5:**
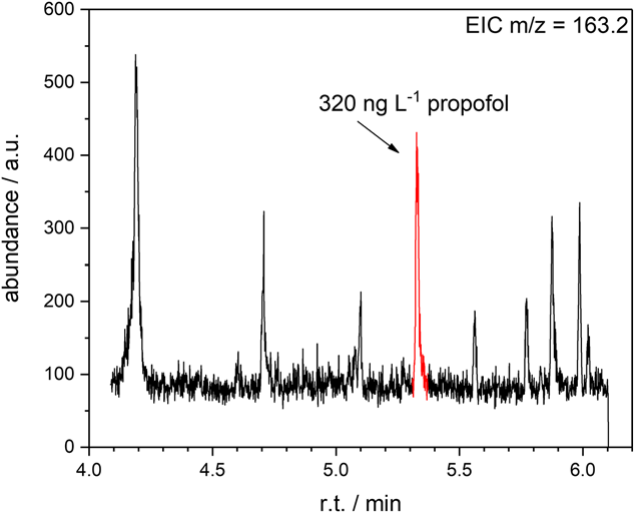
Chromatogram of a cerebral microdialysate sample containing 320 ng/L propofol measured with the developed DI‐SPME–GC–MS method

The presented new DI‐SPME–GC–MS method for the reliable and highly sensitive detection of propofol in cerebral microdialysate samples was proven to be robust, reproducible, and reliable over a broad range of concentration levels (50 ng/L to 200 μg/L). The lower LOD was determined to be 50 ng/L (S/N = 3). During method development, no recovery related issues or carryover phenomena were encountered, since extraction efficiencies were included in the calibration, and thermal desorption was quantitative. A precision of 2.7% RSD between five consecutive measurements and an interday precision of 6.4% RSD could be observed. Additionally the developed DI‐SPME–GC–MS method showed good linearity (*R* ^2^ ≥ 0.9999) in a range between the lower LOD (50 ng/L) and 200 μg/L. In this context, Figure 5 shows a chromatogram of a cerebral microdialysate solution containing 320 ng/L propofol obtained from a patient receiving 83.33 μg kg^−1^ min^−1^ of propofol intravenously. The corresponding sample was a leftover obtained during routine clinical measurements.

### 3.2 Recovery of cerebral microdialysis regarding propofol

Following the theoretic approach stated above, it is easily feasible to calculate the extraction fraction parameter$\;E_{d}$:

Ed=cp−cince−cin=143 ng /L−0 ng /L1000 ng /L−0 ng /L=1−exp−1QdRp+Rm+Re=0.143

$$\;\frac{c_{p}}{E_{d}}=\frac{c_{p}}{0.143}\;=\;c_{e}$$

Since $c_{P}$ can be measured using the above‐described DI‐SPME–GC–MS method, it is possible to calculate the analyte concentration in the extracellular fluid $c_{e}$ within the brain.

Considering that $Q_{d}$ = 0.3 μL/min in clinical use as recommended by the Ungerstedt et al. 15, the use of Equation (2) leads to:

Rp+Rm+Re=R=21.6 min /μL

Since it is generally assumed that the mass transfer resistance is primarily resulting from the resistance implied by the membrane 33, simplification leads to:

$$R_{m}>>R_{e,\;p}$$

R=Rm=21.6 min /μL

Based on this simplification, it can be assumed that recovery experiments conducted in vitro can be used to determine in vivo recoveries as well.

## 4 CONCLUDING REMARKS

The developed DI‐SPME–GC–MS method was proven to be a reliable and highly sensitive option for the analysis of propofol in human cerebral microdialysate samples of low volumes. The method development included especially the optimization of extraction/preconcentration times, to be both sensitive and time efficient. The determination of the precision of the method in interday and intraday experiments showed satisfying results, expressed as sufficiently low RSDs even without the use of internal standards and with standard solutions around the lower LOD. Linearity was confirmed in a broad range, between the lower LOD (50 ng/L) and 200 μg/L, enabling the analysis of microdialysate samples from intensive care patients receiving propofol as a part of total intravenous anesthesia.

Although the presented method is rather time consuming in terms of sample preparation respectively preconcentration, it is rewarding because of the unique low LOD and the satisfying linearity and reproducibility and is moreover easy in terms of the small number of preparative steps. Considering additionally the invasive character of cerebral microdialysis, for which reason the number of patients, and therefore, the number of samples is limited, the increased time required for a single analysis is not a serious disadvantage.

Furthermore the permeability of propofol through the commercially available 71 High Cut‐Off Brain Microdialysis Catheter was proven and quantified, allowing the application of this system in clinical research projects targeting the cerebral pharmacokinetics of propofol, and adequate calculation of extracellular fluid concentrations.

Since the levels, determined with DI‐SPME–GC–MS and adjusted for the extraction efficiency of the microdialysis, represent the unbound drug concentration available for the surrounding cells, highly interesting cerebral pharmacokinetic data become available. Consequently, this may be a first step to assess the quality of the target controlled infusion models of Marsh and Schnider, based on the quantitation of propofol at the target site, the living human brain.

## CONFLICT OF INTEREST

The authors have declared no conflict of interest.
